# Costs and effectiveness of the supervision, performance assessment and recognition (SPARS) strategy for medicines management in Uganda

**DOI:** 10.1186/s40545-019-0196-8

**Published:** 2019-12-03

**Authors:** Brendan Kwesiga, Anita Katharina Wagner, Morries Seru, Dennis Ross-Degnan, Birna Trap

**Affiliations:** 1 USAID/Uganda Health Supply Chain Program, Management Sciences for Health, Plot 15, Princess Anne Drive, Bugolobi, P.O. Box 71419, Kampala, Uganda; 2 Harvard Medical School and Harvard Pilgrim Health Care Institute, 401 Park Drive Suite 401, Boston, MA 02215 USA; 3 Ministry of Health, Pharmacy, Division, Lourdel Road, Wandegeya, Kampala, Uganda

## Background

Essential medicines and health supplies (EMHS) are fundamental to providing quality health care that is critical for saving lives. Ensuring that medicines of good quality are available, accessible, affordable, and appropriately used is a key objective of the Uganda *National Medicines Policy* [1]. Central to achieving this objective are good medicines management practices at all health facilities. Despite Uganda’s long-standing commitment to its medicines policy, the pharmaceutical supply chain has faced many well-documented constraints [2–5].

### Uganda’s health system

Uganda’s estimated population was 38.8 million people as of 2018. The population size is growing at an average annual rate of 3%, which is one of the highest in the world [6]. Communicable diseases remain the leading cause of years of life lost and result in 48% of the mortality in Uganda; in addition, non-communicable diseases are becoming increasingly important as a cause of morbidity and mortality [7].

In 2013, Uganda had 116 administrative districts with 6404 health facilities. Of these 48% were government-owned, 15% were private not-for-profit, and the rest (37%) private for-profit [8]. The public sector consisted of government and private not-for-profit facilities and included two national referral hospitals, 16 regional referral hospitals, 117 general hospitals, 196 health centers (HC) level IV, 1291 HCIII and 2354 HCII facilities. EMHS for government facilities are supplied through the government-owned National Medical Stores [7]. The private not-for-profit facilities are supplied through the Joint Medical Stores. Medicines are supplied free of charge to patients in government facilities.

In fiscal year 2015/16 the total government budget allocated to EMHS in government facilities was US$94 million with per capita government sector EMHS expenditures of only US$0.80 (excluding antiretroviral treatment, tuberculosis, and malaria medicines) [9]. When we consider the external donor budget for antiretroviral treatment, tuberculosis, malaria medicines and some essential medicines for the same year (2015/16), per capita EMHS expenditures were US$1.90 [10]. In view of severe governmental resource constraints and substantial donor investments (close to 80% of total medicines expenditures in 2015/116) [9], strengthening medicines management is crucial for maximizing scarce resources and limiting waste.

Medicines management processes include quantification, procuring, storage, stock management, prescribing, dispensing, ordering and reporting. These processes are complex and often interlinked and their implementation must be well managed to achieve optimal outcomes. Successful implementation of good medicines management practices requires adequate regulatory, financial, and human resources including facility staff with the appropriate skill set and relevant information [11].

In an effort to improve medicines management capacity at health facilities, Uganda developed and implemented a multi-pronged, evidence-based supervision, performance assessment, and recognition strategy (SPARS) [12]. SPARS combine supervision with indicator-based performance assessment to identify problems, track improvements and includes facility recognition schemes. Performance is assessed in five domains (ordering/reporting, stock and storage management, prescribing, and dispensing) using 25 indicators. Medicines management supervisors (MMS) visit facilities to assess staff performance using pre-defined data collection forms. Based on the assessment the MMS identify areas in need of improvement, and they support facility staff in implementing change. MMS then enter SPARS scores into the online pharmaceutical information portal (PIP).

The information generated through SPARS guides strategic decisions to improve access, ensure availability, and encourage appropriate use of EMHS [13]. SPARS has improved performance in all medicines management domains across all levels of care [14].

Whereas the benefits of SPARS in improving medicines management are well documented [14], we wanted to estimate the costs and cost effectiveness of SPARS implementation in public (government and private not-for-profit) health facilities in Uganda. This information is critical for further SPARS scale up in Uganda and for SPARS implementation in countries with similar contexts that want to consider rolling out SPARS as a national strategy.

## Methods

### SPARS intervention

SPARS has been implemented by Uganda’s Ministry of Health since 2010 with support from the US Agency for International Development. SPARS is implemented by district-level health care staff who are trained as MMS to provide on-the-job supervision and training of health workers. Components of the SPARS program are summarized below and detailed elsewhere [12].

### Components of the SPARS program

#### MMS selection

The district health officers (DHOs) select three to five MMS in their districts depending on the number of health sub-districts. One district-level MMS supervises hospitals and HCIV facilities, and one to four health sub-district-MMS supervise lower-level health facilities in the health sub-districts. MMS are health workers who are already employed within the district health system; MMS can be clinical officers, nurses, midwives, pharmacy staff, or storekeepers that implement SPARS along with other duties; the supervisory task is an added responsibility. The DHO monitors performance of MMS with oversight from regional pharmacists and the Ministry of Health’s Pharmacy Department.

#### Equipment provision

To facilitate the performance assessment and ensure high-quality data, each MMS is provided with a computer (netbook) and a modem for electronic data submission. To enable MMS to travel to the facilities they supervise, they are provided with a motorcycle (175 cc, 4 stroke) and appropriate riding gear including helmets and boots. The motorcycle is meant to be used exclusively by the MMS to ensure long life, although the MMS might use the motorbike for other work-related business.

#### MMS capacity building

To become qualified, MMS have to undergo several SPARS trainings that include a theoretical training, a practical field orientation, defensive motorbike training, computer training, and data use training. The theoretical training is a two-week examinable residential workshop. The workshop covers medicines management, problem-solving, communication, mentoring, assessing performance using SPARS indicators, and work planning and reporting. To ensure quality and sustainability, the training is implemented by faculty from Makerere University. Next comes a five-day in-service supervision of the MMS implemented by experienced MMS in the trainees’ district or in nearby districts. MMS then take part in a three-day residential workshop on the use of computers and data entry and use. Each MMS finally receives a six-day residential course on defensive motorcycle riding, repairs, and licensing implemented by professional motorbike drivers and mechanics. The MMS are provided with supervision tools such as an EMHS manual, stock books, a supervisory book to record and track assessment findings and agreed tasks and laminated job aids. A white board displaying a spider graph that depicts and tracks each facility’s SPARS implementation and performance is also provided.

#### Number of visits

In addition to supervising higher-level facilities, the district MMS also have the responsibility for mentoring and supporting the health sub-district MMS. District and health sub-district MMS are expected to make three and five supervisory visits per month, respectively, and each health facility should be supervised about five times within the first year of SPARS. After five visits, the facility staff should have built the skills required to ensure good medicines management, and subsequent supervisions are focused on maintaining these skills. After finishing the initial five visits to their assigned facilities, MMS can start taking on other tasks. These tasks include supervising pharmaceutical financial management or antiretroviral management, redistribution of supplies, and supporting medicines therapeutic committees. All tasks, that are relevant for strengthening medicines management beyond SPARS.

#### MMS facilitation

Following each supervisory visit, the MMS submits his or her SPARS report into the PIP, an online information system. The MMS is then eligible to receive a daily travel allowance and transport allowance enough to cover fuel, minor repairs, and oil changes. The payment is made using mobile money following each visit documented through the SPARS report submission.

#### Repair and maintenance

To ensure that motorcycles and computers remain functional to support SPARS implementation, routine repair and maintenance are critical. The DHO receives funds for annual motorcycle service and maintenance, insurance, repairs, and a set of new tires annually per motorbike. The funds for motorcycle maintenance and repair are linked to a minimum number of MMS supervision visits implemented and documented with reports.

### Communication, coordination, and collaboration

To ensure coordination and collaboration among the DHOs, MMS, other Ministry of Health staff, and community partners in SPARS implementation, the Ministry of Health organizes quarterly implementing partner and district coordination meetings. The Ministry of Health also disseminates district, regional, and national SPARS performance reports to monitor the performance of MMS and to share lessons learned [13]. The DHO receives airtime and telephone-time to coordinate and oversee SPARS implementation in the district. The MMS also receive airtime and internet-time for communication and data submission.

#### SPARS information use

To facilitate evidence-based decision making for improving medicines management, all SPARS reports are collected centrally and stored in the PIP. To improve the use of SPARS data, all MMS and DHO have been trained in accessing and using the PIP and generating reports based on SPARS data.

#### Recognition scheme

Health facilities that meet performance goals receive items such as tea and sugar, mugs, pens, a wall clock, T-shirts, and calendars. During SPARS implementation, health facilities were also provided some items that the Ministry of Health should have provided, including thermometers, stock books, and shelves to store EMHS.

### Measurement of costs

Through this study, we intended to answer two questions: (1) *What does it cost per year to implement and operate SPARS as a national strategy?* and (2) *What were the SPARS-related costs for a health facility to attain an adequate SPARS score?*

We answered the first question by calculating the costs to implement and run SPARS for three years per facility between 2011 and the end of 2014. The costs captured were those incurred to strengthen medicines management and are incremental to the costs of routinely supplying medicines in existing systems. Although during SPARS implementation, health facilities were provided with standard pharmacy equipment such as tools for medicines management (stock books, stock cards, borrow and lend records, expired medicines registers, EMHS list, EMHS manual, clinical guidelines, tablet counting trays, plastic dispensing bottles, measuring beakers, spatulas, dispensing envelopes, shelving units, and thermometers). We did not include the cost of these items in the SPARS cost estimate because they should be supplied as part of standard system operations. We chose a conservative approach by including the full cost of capital items (computers, motorbikes, service and maintenance) even though they might be used for other purposes beyond SPARS. We annualized all capital costs based on their expected useful life and applied a discount rate of 3% to obtain their annual depreciation value. We estimated the costs of data management by including the cost of the MMSs’ computers, modems, air- time, and MMS training in data entry and use. We did not include the salaries for the DHO, regional pharmacists, and district staff working part time as MMS because these staff already have supervision and oversight as part of their job descriptions. However, SPARS has made supervisory visits more regular and effective. The cost of oversight and supervision of MMS by district, regional and central level staff have been included. Costs for program personnel^1^ and Pharmacy Department staff to design, coordinate, and implement SPARS have been excluded as it is part of existing duties and salaries.

We used information from the SPARS program staff, program reports, expenditure records, and invoices from 2011 through 2014 to identify activities and resource inputs used to implement SPARS. We used 2016 prices to value the resource inputs and estimate the costs because those were readily and completely available. Thus, no inflation adjustment was done. We collected data in both Uganda shillings (UGX) and United States (US) dollars based on the currency used to procure inputs and implement activities. In the analysis and presentation of results, cost data is presented in 2016 US$ using an average exchange rate between 2011 and 2014 of UGX 3500 to US$1.

To answer the second question, we calculated the SPARS effectiveness as the cost for a health facility to reach adequate SPARS score.

### Measurement of intervention effectiveness

Measurement of SPARS effectiveness has been described before [12, 14]. For this study, we used data from the PIP database and selected 1460 facilities that started SPARS implementation in 2011 or 2012 and followed each facility for three years to assess SPARS effectiveness. When a health facility reached 75% of the maximum SPARS score—18.75 of 25 points—during the three observation years, its medicines management practices were deemed effective. In general, a facility that achieves a 75% SPARS score is performing adequately in the SPARS domains of dispensing quality, prescribing quality, stock management, storage management, and ordering and reporting.

### Measures of SPARS cost-effectiveness

As the purpose of SPARS is to improve medicines management at health facilities, the cost-effectiveness analysis aimed to determine the costs for a health facility to attain an adequate SPARS score. To assess SPARS cost effectiveness, we estimated the cost per health facility to achieve a SPARS score ≥ 18.75 over the three-year observation period per facility. We divided the total incremental cost of SPARS implementation over the follow-up period (three years for each of 1460 facilities) by the number of facilities that attained the desirable SPARS score over the same period (899). We excluded the five facilities having an adequate score at baseline. Total costs were incurred in four calendar years (2011, 2012, 2013, and 2014) because facilities were enrolled in 2011 and 2012 and then followed for three observation years within four calendar years, and then divided by three to determine an annual cost.

### Sensitivity analyses

To assess whether the estimated incremental cost-effectiveness ratio (ICER) is robust to changes in either cost or effectiveness calculations, we carried out sensitivity analyses. With regards to costs, we varied potential cost drivers by including costs of the standard pharmacy equipment, costing different approaches to MMS training (base case is using a training institution versus in-house training), and assuming there is already capacity among MMS in computer use and motorcycle riding.

We assessed the robustness of the ICER to change in effectiveness based on a ± 10% change in the number of facilities attaining an adequate SPARS score. We also estimated the best-case scenario (based on the lowest cost and highest effectiveness) and worst-case scenario (based on the highest cost and lowest effectiveness) within the plausible ranges considered for the sensitivity analysis.

## Results

This section presents the costs, effectiveness, and the cost effectiveness following SPARS implementation between 2011 and 2014. To link cost and effectiveness, we estimate the incremental cost effectiveness ratio. We also present results of the sensitivity analysis.

### Costs

Table 1 shows the annual costs of implementing SPARS in 1460 facilities between 2011 and 2014. In total, the 1460 facilities received 7616 supervisory visits (i.e., about five visits per facility) over the three-year per facility follow-up period. The 264 MMS who made the supervisory visits were fully trained and equipped.

**Table 1:**
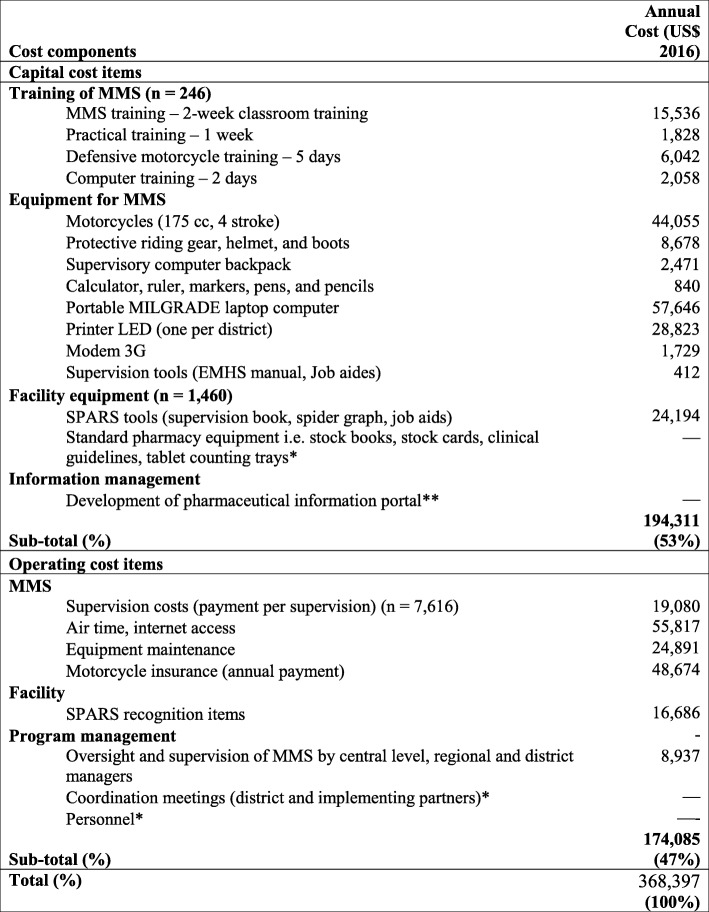
Annualised economic cost of establishing and implementing SPARS

*Costs covered within existing duties, salaries or by government ordinary expenses

**Although funded by the donor, PIP development cost was not included in the calculations as the SPARS does not depend on PIP

The total cost of implementing SPARS in 1460 facilities over three years was US$1,105,190, while the annual cost was US$368,397. The capital/establishment costs accounted for 53% and operating costs for 47% (Table 1).

As noted, SPARS implementation relies on using MMS who are government employees within their districts and undertake medicines management supervision as an additional responsibility. Therefore, labor costs are covered within their existing government salaries.

### Effectiveness

Table 2 shows the effectiveness of SPARS in the 1460 health facilities that received supervisory visits for three years between 2011 and 2014. At baseline (visit 1) the median SPARS score across all health facilities was 10.3. Over 60% (904/1460) of the facilities attained and maintained an adequate SPARS score of ≥18.75 during that time; five facilities had already reached the target score at baseline. Therefore, we assessed the incremental effectiveness as 61.6% based on 899 facilities that increased their inadequate scores at baseline over the course of the three years of SPARS supervision.

**Table 2:**
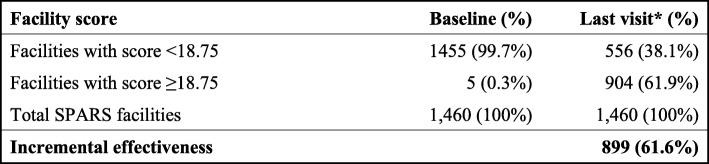
Effectiveness of SPARS following three years of SPARS implementation

*Last visit within a three-year follow-up period per facility

The 1460 facilities received 7616 supervisory visits, with a median of six visits per facility and a range of 1–12 visits over the three-year period. The 904 facilities with an adequate score at their last visit received 4227 visits with a median of five visits and a range of 1–12 visits.

### Cost effectiveness of SPARS

Table 3 presents the total cost associated with implementing SPARS for medicines management within an existing health system and the additional effectiveness due to implementing SPARS. The results show that the incremental cost for every additional facility attaining the desirable SPARS score is equal to US$1229.

**Table 3:**
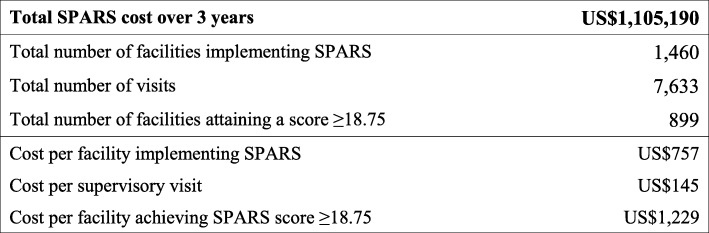
SPARS cost-effectiveness ratios

### Sensitivity analysis

**Fig. 1:**
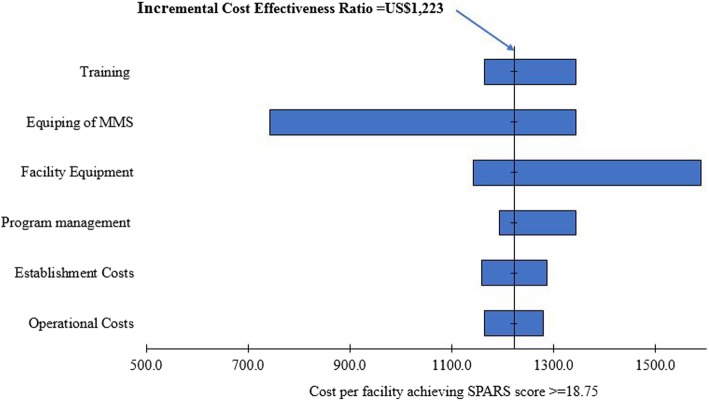
One-way sensitivity analysis comparing effect on cost per facility achieving SPARS score due to variation of cost parameters

We assessed how the estimated ICER varied with changes to parameters that influence the cost of SPARS. Figure 1 shows that the biggest cost driver is the investment in equipping the MMS. The most cost-effective scenario (i.e., variation that produces the lowest ICER) is when we assume that the MMS do not have to be equipped. The least cost-effective approach (highest ICER) is when health facilities have to be provided with all standard equipment for properly supplying medicines, including shelving.

The combined variation of both cost and effectiveness within a defined range (number of facilities attaining satisfactory score +/− 10%) shows that the SPARS ICER ranges from 2016 US$1000 (best case) to US$1494 (worst case) (Table 4).

**Table 4:**
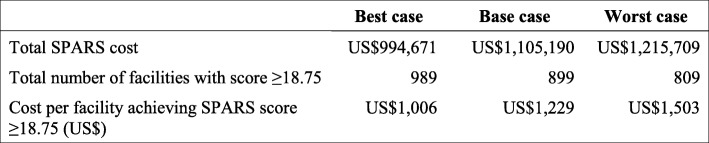
Sensitivity analysis scenarios

## Discussion

The study assessed the cost and cost effectiveness of implementing SPARS, which is a multi-pronged intervention to improve medicines management practices at facility level. Evidence shows that SPARS is an effective intervention to improve performance in key medicines management domains [14]. However, to implement a system-strengthening intervention, knowing the cost and potential return on the investment is important. In this study, we not only show what it costs to implement SPARS, but also the costs for a health facility to reach an adequate SPARS performance. To our knowledge, this is the first study to estimate costs and cost effectiveness of a comprehensive supportive supervision strategy to improve medicines management in a health system. A previous study that looked at the cost-effectiveness analysis of a supportive supervision intervention - strengthening immunization service delivery in India found, that it would cost US$3091 to obtain a 1% increase in health facility immunization performance scores [15].

How can we judge whether investing in SPARS to improve facility medicines management is worthwhile? Leech and colleagues have urged that the decision on whether an intervention (such as SPARS) is cost effective should reflect the health system context, challenges, and priorities [16]. Availability of medicines within a health system is the foundation for improving population health outcomes. When medicines are not well managed, products expire or spoil, medicines shortages occur, and patients receive no medicines, poor quality medicines, or inappropriate medicines [17]. It has also been noted that improving supply chain management by increasing efficiency and effectiveness is critical in reducing the global burden of disease, especially in resource-limited settings [18]. In fiscal year 2015/16, the Government of Uganda allocated 18% of the total health budget to medicines and medical supplies (which is the second largest line item after personnel/human resources expenses). Although this proportion is significant, the actual amount allocated in real terms is sub optimal. The existing per capita allocation for medicines of $2 result in sub-optimal availability of medicines even with significant donor funding [9]. Given enormously limited resources available for medicines procurement, it is critically important to optimize these resources and minimize waste. SPARS provide the system foundations to manage scarce medicines resources well.

While this study does not directly link better medicines management with savings from reduced waste and better health outcomes, others have shown that supply chain strengthening can reduce stock outs and increase medicines availability [19]. Seidman and Atun recommend that policy makers “examine the root causes of inefficiencies in pharmaceutical supply chain and procurement processes to determine how best to improve health systems performance”. In 2010, the Ugandan Ministry of Health conducted a policy options analysis [20], which was informed by a baseline assessment of root causes of inefficiencies that gave rise to the design and implementation of SPARS [21]. Having previously shown SPARS effectiveness [14], our paper details the costs of SPARS to achieve the desired effect.

If investing in SPARS is considered worthwhile to facilitate effective and efficient use of the Ugandan government’s annual EMHS budget, can the Ugandan government afford it? In 2015/16 the government spent US $94 million on EMHS [10], while donors spent over US$200 million [9]—mainly on medicines for HIV, tuberculosis, and malaria. Based on our estimates from this study, implementing and operating SPARS costs about US$370,000 annually for 1460 facilities, which would extrapolate to approximately US$760,000 for about 3000 government sector facilities or about 0.3% of the total government- and donor-funded EMHS budget.

Donors and development partners may want to contribute to SPARS implementation because SPARS provides facility staff the basic medicines management skills. These skills are critical for managing all medicines, including antiretrovirals and other high-cost medicines, for strengthening pharmaceutical information systems, and for managing pharmaceutical finances to assure efficient use of donor resources. In Uganda, we also found that MMS contributed to health system objectives beyond SPARS, such as shifting from a kit- to an order-based EMHS distribution system, applying cost recovery mechanisms, and facilitating the introduction of new clinical guidelines. Donor and country investment in pharmaceutical supply chain systems seems a prerequisite for effective and efficient use of development funds and will form a foundation for stronger health systems, including the ability to address emerging challenges, such as non-communicable diseases [11].

### Limitations

We only observed facilities for three years, which restricts our knowledge of longer-term costs and effectiveness. Further, the investment required for implementing SPARS for the first time will depend on how rudimentary the basic structures and processes of a country’s health and pharmaceutical systems are; some may need to make investments that Uganda already had in place, such as human resources and a health facility supervisory infrastructure. In addition, the potential effectiveness of SPARS will depend on facility performance at the initial visit [14]; a country could decide to implement SPARS only in poorly performing facilities.

## Conclusion

While several studies document the effects of interventions based on supportive supervision, documentation of the costs and cost-effectiveness of such interventions are limited. We document the costs and cost effectiveness of a strategy that has been shown to improve medicines management performance in Uganda. Without sound and transparent EMHS management that enables affordable access to medicines, low-income countries such as Uganda will not be able to achieve the goals of universal health coverage. Putting in place systems to manage medicines efficiently will optimize the use of funds and lead to the sustainable financing that is a critical component of a health system that provides coverage to all.

## Data Availability

Data analyses, and other materials can be obtained upon request from the corresponding author.

## Abbreviations

DHO: District health officer
EMHS: Essential medicines and health supplies
HC: Health centers
ICER: Incremental cost effectiveness ratio
MMS: Medicines management supervisors
PIP: Pharmaceutical Information Portal
SPARS: Supervision Performance Assessment and Recognition Strategy
UGX: Uganda shillings
US: United States
USAID: United States Agency for International Development

## Footnotes

1 The USAID-funded Securing Ugandans Right to Essential Medicines program led SPARS implementation with the Ministry of Health.
